# Comparison of safety and haemodynamic performance between the Avalus™ stented aortic valve bioprosthesis and Magna™ valve in Japanese patients

**DOI:** 10.1007/s11748-020-01566-1

**Published:** 2021-01-05

**Authors:** Naoki Tadokoro, Satsuki Fukushima, Yusuke Shimahara, Tetsuya Saito, Naonori Kawamoto, Takashi Kakuta, Kimito Minami, Tomoyuki Fujita

**Affiliations:** 1 Department of Cardiovascular Surgery, National Cerebral and Cardiovascular Center, 6-1 Kishibeshimmachi, Suita, Osaka 564-8565 Japan; 2 Department of Surgical Intensive Care, National Cerebral and Cardiovascular Center, Suita, Osaka Japan

**Keywords:** Aortic valve replacement, Bioprosthetic valve, Japan, Avalus

## Abstract

**Objectives:**

A new stented bovine pericardial valve (Avalus™) has been proven safe and effective with good hemodynamic performance in Western populations. However, its use in Japanese patients is poorly understood. We retrospectively compared the feasibility, safety, and valve haemodynamics between the Avalus™ and Magna™ valves in patients who underwent surgical aortic valve replacement (SAVR).

**Methods:**

This study included 87 patients receiving an Avalus™ valve and 387 receiving a Magna™ valve. We evaluated adverse events, outcomes, and valve haemodynamics within 1 year postoperatively. There were no significant differences in any surgical risk scores.

**Results:**

No in-hospital mortality occurred in the Avalus™ group, but two mortality events occurred in the Magna™ group. No pacemaker implantation for complete atrioventricular block was required in the Avalus™ group. There was no significant difference in in-hospital or clinical outcomes between the two groups until 1 year postoperatively. Left ventricular mass index reduction appeared to predominate in the Avalus™ over Magna™ group. There was no significant difference in the mean pressure gradient or effective orifice area of each valve size at 1 week or 1 year between the two groups, apart from the mean pressure gradient of the 23-mm valve at 1 week. Three patients (3.4%) in the Avalus™ group and 39 (10.8%) in the Magna™ group (*p* = 0.12) had severe patient–prosthesis mismatch at 1 week postoperatively.

**Conclusions:**

The new Avalus™ stented aortic valve bioprosthesis was associated with good in-hospital outcomes and good valve functionality post-SAVR in Japanese patients.

## Introduction

Surgical aortic valve replacement (SAVR) using a stented bioprosthetic valve is the standard treatment for severe aortic valve stenosis and insufficiency. Many types of stented bioprosthetic valves with bovine pericardium are available, such as the Carpentier–Edwards Perimount Magna™ or Magna Ease™ (Edwards Lifesciences, Irvine, CA, USA), which have been repeatedly upgraded to enhance their functionality, implantability, and durability. A new bovine pericardial stented valve was recently developed and marketed as the Avalus™ valve (Medtronic, Minneapolis, MN, USA). This new valve is characterised by its short stent post and large cuff, which enhance implantability, and by its pliable stent post, which reduces shear stress during diastole, potentially leading to enhanced durability [1]. Clinical studies of this new valve confirmed its feasibility and safety in addition to its good 1-year functionality; however, the institutions included in these studies were located in Western countries, where patients’ body size and thus aortic annulus size are generally larger than those in Asian populations [2]. In this study, we reviewed our first 87 patients who underwent SAVR using the Avalus™ valve and investigated the feasibility, safety, and valve haemodynamics of the Avalus™ valve in Japanese patients. We compared these patients with those who underwent SAVR using the Magna™ valve, which was the standard prosthesis prior to the Avalus™.

## Methods

### Study cohort and data collection

This was an observational, single-centre cohort study. The institutional surgical database contained a consecutive series of 769 patients who underwent SAVR with a biological prosthetic valve at the National Cerebral and Cardiovascular Center from April 2012 to March 2020. Of these 769 patients, the Avalus™ prosthesis was used in 102 (13.2%) and the Magna™ valve was used in 419 (54.4%). After excluding patients who underwent concomitant mitral valve replacement, the remaining 87 patients with an Avalus™ valve and 381 patients with a Magna™ valve were enrolled in this study. We reviewed the patients’ medical charts, surgical reports, and referral letters to collect the study data. Major adverse cardiac and cerebrovascular events (MACCE) were classified according to the standardised definitions [3]. Patient follow-up was completed at the end of the study in 87 patients (100%) in the Avalus™ group and in 362 patients (95.3%) in the Magna™ group (*p* = 0.032), with a follow-up of 16.0 months (interquartile rang (IQR), 5.5–20.0) and 74.0 months (IQR, 47–113), respectively (*p* < 0.01). MACCE were classified as occurring within 1 year postoperatively. Data collection was performed in April 2020. Preoperatively, all patients provided written informed consent for surgery and the use of their data for diagnostic and research purposes. This study was approved by our institutional review board (approval number: M30-026).

### Patient backgrounds and characteristics

There were several significant differences in the backgrounds and characteristics between the two groups (Table 1). There were significant differences in background factors such as hypertension, HbA_1_C, and kidney function, the New York Heart Association functional class, valve pathology. In contrast, the body surface area and body mass index, the presence of a bicuspid valve, history of cardiac surgery were not significantly different between the two groups. As a result, there were no significant differences in any surgical risk scores.

**Table 1:** Patients’ characteristics

	Avalus	Magna	*p* value
	*n* = 87	*n* = 381	
Age (years)	73 [66, 79]	70 [64, 76]	0.022
Male	50 (58.1)	231 (60.6)	0.761
Body surface area (m^2^)	1.62 [1.47, 1.75]	1.62 [1.49, 1.75]	0.943
Body mass index (kg/m^2^)	22.7 [14.6, 35.4]	22.8 [13.3, 32.6]	0.148
Valve pathology			
Aortic stenosis	41 (47.3)	240 (63.0)	0.001
Aortic regurgitation	36 (41.4)	93 (24.4)	0.005
Mixed	5 (5.7)	24 (6.3)	0.90
Infective endocarditis	3 (3.4)	5 (1.3)	0.84
Prosthetic valve failure	1 (1.1)	15 (3.9)	0.335
Prosthetic valve endocarditis	1 (1.1)	4 (1.1)	1
Bicuspid aortic valve comorbidity	25 (29.4)	120 (33.2)	0.583
Coronary stenosis	13 (14.9)	76 (19.9)	0.357
Atrial fibrillation	19 (22.1)	60 (15.7)	0.208
Hypertension	55 (63.2)	285 (74.8)	0.04
Hyperlipidemia	40 (46.0)	190 (49.9)	0.592
Diabetes	12 (13.8)	66 (17.3)	0.524
HbA1c (%)	5.8 [5.5, 6.1]	5.5 [5.2, 5.9]	< 0.001
Smoking	36 (41.4)	135 (35.4)	0.36
Carotid stenosis	2 (2.3)	10 (2.6)	1
Chronic kidney disease	8 (9.2)	22 (5.8)	0.351
Dialysis	7 (8.0)	8 (2.1)	0.012
Creatinine (mg/dl)	0.91 [0.73, 1.14]	0.85 [0.67, 1.01]	0.019
BNP (pg/ml)	120 [54, 317]	102 [44, 264]	0.257
New York Heart Association class			< 0.001
I	22 (25.3)	22 (5.8)	
II	55 (63.2)	321 (84.3)	
III	10 (11.5)	33 (8.7)	
IV	0 (0.0)	5 (1.3)	
Risk score			
Euro SCORE II	1.8 [1.3, 2.9]	1.6 [1.0, 3.2]	0.613
Japan score	2.0 [1.4, 4.2]	2.0 [1.3, 3.5]	0.626

Data are presented as median (interquartile range) or number (%). HbA1c, haemoglobin A1c; BNP, brain natriuretic peptide; EuroSCORE, European System for Cardiac Operative Risk Evaluation; Japan SCORE, Japan System for Cardiac Operative Risk Evaluation

### Surgical indications, procedure, and postoperative care

The surgical indications for SAVR were determined by the institutional cardiac team according to current clinical guidelines [4] [5] (Table 1). The surgical approach, either median sternotomy or a minimally invasive cardiac surgery (MICS) approach such as partial sternotomy or right mini-thoracotomy, was determined by discussion among the surgical team (Table 2). The MICS approach was selected in relatively young patients without severe atherosclerotic change in the aorta and undergoing isolated SAVR. All valves were placed in the supra-annular position by non-everting mattress sutures. Selection of the bioprosthetic valves was modified according to the era of surgery. From April 2012 to July 2018, the Magna™ valve was the primary choice, but after August 2018, the Avalus™ valve was the primary choice. However, other prostheses, including mechanical valves, were also used during the study period at the discretion of the surgeons and/or patients. In our institute, a bioprosthetic valve was indicated in patients aged ≥ 60 years and those for whom a mechanical valve was not indicated medically and/or socially.

**Table 2:** Intraoperative variables

	Avalus	Magna	*p* value
	*n* = 87	*n* = 381	
Procedure			
Isolated AVR	41 (47.1)	204 (53.5)	0.336
MICS procedure	12 (13.8)	22 (5.8)	0.018
Right thoracotomy	12 (13.8)	11 (2.9)	< 0.001
Partial sternotomy	0 (0.0)	11 (2.9)	0.365
Concomitant procedure			
Ascending aorta surgery	9 (10.3)	37 (9.7)	1
Coronary artery bypass grafting	12 (13.8)	78 (20.5)	0.202
Mitral valve repair	11 (12.6)	25 (6.6)	0.09
Tricuspid valve repair	6 (6.9)	12 (3.2)	0.185
Maze procedure	11 (12.6)	32 (8.4)	0.303
Myectomy	3 (3.4)	10 (2.6)	0.952
Nicks procedure	2 (2.3)	7 (1.8)	1
Operation time (min)	264 [217, 315]	279 [236, 341]	0.017
Aortic cross-clamp time (min)	90 [74, 114]	89 [72, 113]	0.352
Cardiopulmonary bypass time (min)	128[105, 161]	133[107, 163]	0.57
Prosthesis size (mm)			0.485
19	17 (19.5)	88 (23.1)	
21	23 (26.4)	123 (32.3)	
23	30 (34.5)	98 (25.7)	
25	12 (13.8)	46 (12.1)	
27	5 (5.7)	26 (6.8)	

Data are presented as median [interquartile range] or number (%). AVR, aortic valve replacement; MICS, minimally invasive cardiac surgery

Postoperatively, aspirin at 100 mg/day was prescribed until the last follow-up, and warfarin was prescribed to a target international normalised ratio of 1.5–2.5 for 3 months unless anticoagulant therapy was required (e.g., for atrial fibrillation). Following hospital discharge, the patients were evaluated in the institutional outpatient clinic every 3 months until the last follow-up evaluation.

### Transthoracic echocardiography

All patients were examined by standard transthoracic echocardiography 2 weeks preoperatively and 1 week postoperatively. Forty-five patients (51.7%) in the Avalus™ group and 277 patients (73.3%) in the Magna™ group were again examined by transthoracic echocardiography 1 year postoperatively. The standard parameters were measured. Doppler flow data were acquired from the left ventricular (LV) outflow tract immediately proximal to the prosthesis sewing ring. The modified Bernoulli equation was used to calculate the peak and mean pressure gradient (MPG) across the prosthetic valve. The effective orifice area (EOA) was calculated using a continuity equation on echo Doppler assessments. Severe patient–prosthesis mismatch (PPM) was defined as an indexed EOA (EOA/body surface area) of < 0.65 cm^2^/m^2^ [6]. The LV mass (LVM) was calculated using the following formula: LVM (g) = 0.8 (1.04 ([LV internal diameter in diastole (LVDd) + posterior wall thickness in diastole + interventricular septum thickness in diastole]^3^ − [LVDd]^3^)) + 0.6 and indexed to the body surface area (LMVI). In patients with atrial fibrillation, the MPG was measured as the average of five heartbeats.

### Statistical analyses

Continuous variables are presented as median (IQR) and categorical variables as frequency and percentage. The haemodynamic primary endpoints were the interactions between time points (1 week and 1 year postoperatively) and valve type (Avalus™ versus Magna™) for the MPG, EOA, and LVMI. To determine whether the type of the implanted bioprosthetic valve affected the postoperative adjusted LVMI variation depending on the elapsed time from SAVR, we used a multivariable linear regression model that included a cross-product term between the elapsed time from SAVR and the type of prosthetic valve. To correct for heterogeneous variance and for correlated responses from values measured repeatedly, the Huber–White method was used to adjust the variance–covariance matrix of a fit from least squares [7]. This model was adjusted for the type of implanted bioprosthetic valve, outer diameter of the implanted bioprosthetic valve, age, sex, body surface area, diagnostic differences such as aortic stenosis or aortic regurgitation, and preoperative LVMI. Moreover, if P for time reached statistical significance, a post hoc pairwise comparison between the Avalus™ valve and Magna™ valve was performed. All statistical analyses were performed using two-sided tests at the 5% significance level using R software, version 3.6.0 (www.r-project.org) with the “rms” package.

## Results

### Feasibility of Avalus™ valve for SAVR

Both the Avalus™ valve and Magna™ valve were successfully implanted in all patients. Any patients who underwent surgery by the MICS approach did not require intraoperative conversion to full median sternotomy. Concomitant cardiac procedures were not significantly different between the two groups. Nicks’ annular reconstruction manoeuvre was used in two patients in the Avalus™ group and seven patients in the Magna™ group who had a small aortic annular diameter of < 19 mm. By this procedure, a 19-mm valve was implanted in eight patients; the remaining patient underwent implantation of a 21-mm Magna™ valve. The operation time was slightly but significantly shorter in the Avalus™ than Magna™ group, while neither the bypass time nor cross-clamp time was significantly different between the two groups.

### Safety of Avalus™ valve for SAVR

Although no in-hospital mortality occurred in the Avalus™ group, two patients in the Magna™ group died in-hospital of sustained septicaemia related to infectious endocarditis and by shower emboli related to porcelain aorta, respectively (Table 3). One patient in the Avalus™ group developed a minor ischaemic cerebrovascular accident (National Institutes of Health Stroke Scale score of 2) with a prompt full recovery. In addition, a permanent pacemaker was implanted postoperatively for sick sinus syndrome in one patient in the Avalus™ group. No pacemaker implantation for complete atrioventricular block occurred in the Avalus™ group. As a result, there were no significant differences in in-hospital outcomes between the two groups.

**Table 3:** Clinical outcomes

	Avalus	Magna	*p* value
Survival period (months)	16.0 [5.5, 20.0]	74.0 [47.0, 113]	< 0.001
In hospital MACCEs	2 (2.3)	14 (3.7)	0.75
In-hospital mortality (cardiac)	0	2 (0.5)	
Cerebrovascular accidents	1 (1.1)	4 (1.0)	
Permanent pacemaker implant	1 (1.1)	6 (1.6)	
Heart failure	0	1 (0.3)	
Perioperative myocardial infarction	0	1 (0.3)	
1-year MACCEs	1 (1.1)	9 (2.5)	0.69
1-year mortality	0	2 (0.5)	
Cerebral hemorrhage	0	1 (0.3)	
Unknown	0	1 (0.3)	
Cerebrovascular accidents	0	2 (0.5)	
Permanent pacemaker implant	0	1 (0.3)	
Heart failure	1 (1.1)	2 (0.5)	
Re-intervention due to PVE	0	2 (0.5)	

Data are presented as median [interquartile range] or number (%). MACCEs, major adverse cardiac and cerebrovascular events; PVE, prosthetic valve endocarditis; LVDd, left ventricular internal diameter in diastole; LVDs, left ventricular internal diameter in systole; LVEF, left ventricular ejection fraction; PPM, patient–prosthesis mismatch

All 87 patients (100%) in the Avalus™ group and 375 patients (98.4%) in the Magna™ group were clinically followed-up beyond 1 year postoperatively. No MACCE occurred in the Avalus™ group after discharge from the hospital postoperatively, apart from one patient who presented with congestive heart failure related to atrial fibrillation and was treated in-hospital. There were no significant differences in adverse events between the two groups until the last follow-up.

### Haemodynamic performance of Avalus™ valve

**Fig. 1:**
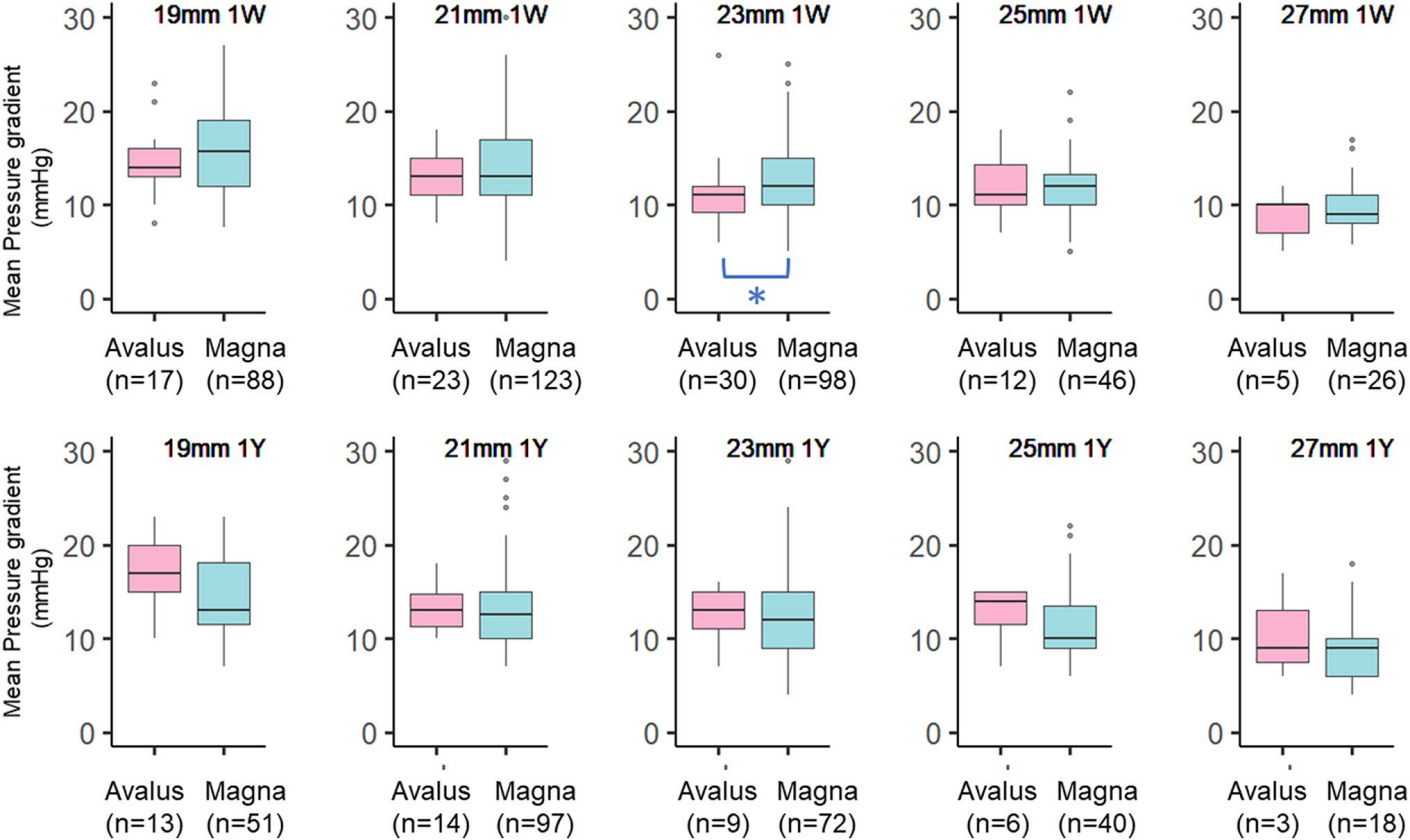
Transthoracic echocardiographic assessment of the mean pressure gradient for each valve

**Fig. 2:**
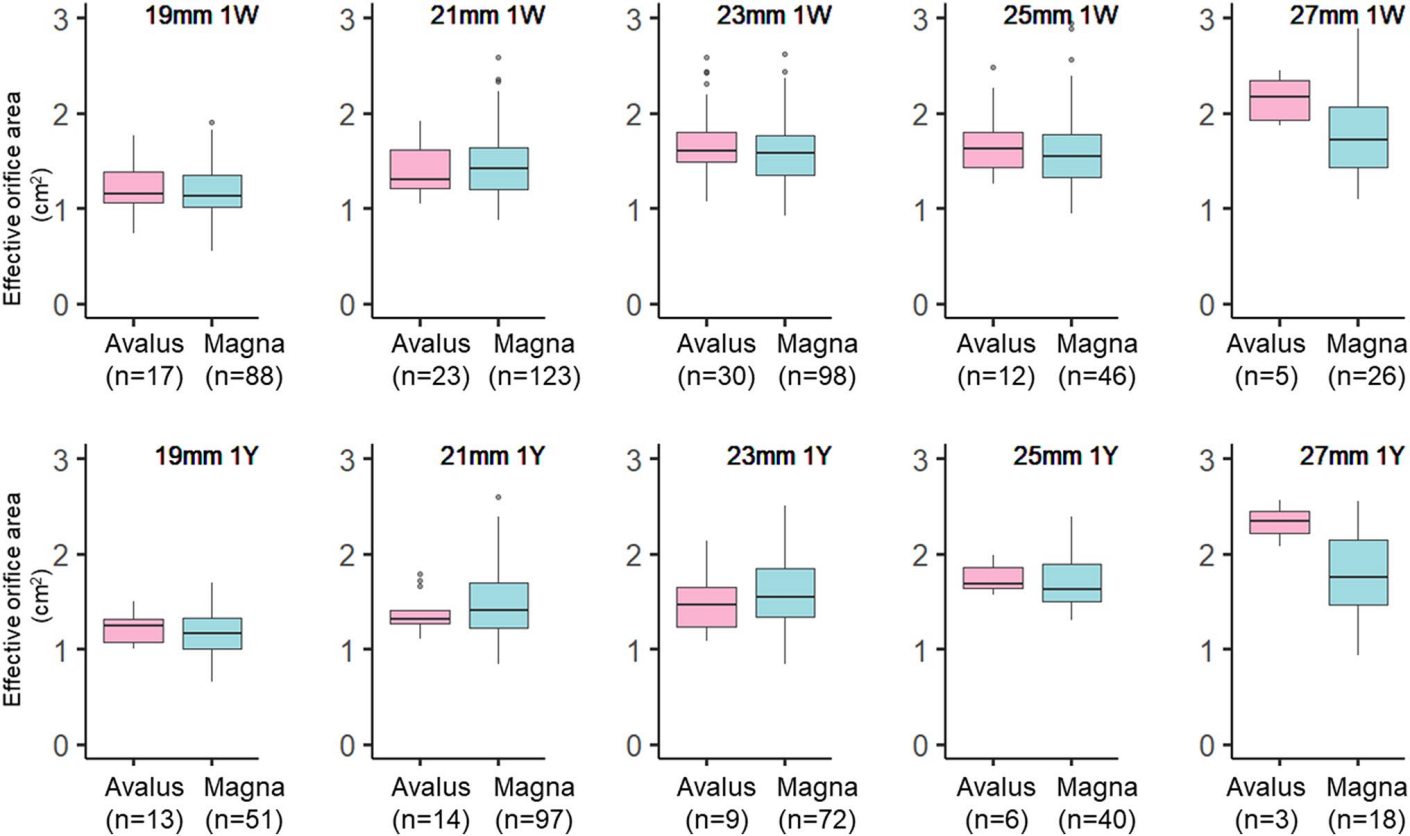
Transthoracic echocardiographic assessment of the effective orifice area for each valve

There was no significant difference in the MPG of each size valve between the two groups at 1 week or 1 year except for the 23-mm valve at 1 week, which showed a significantly higher MPG in the Magna™ group, although the difference was minimal (Fig. 1). There was no significant difference between the two valves beyond 1 year postoperatively. In addition, the EOA for all valve sizes showed no significant difference between the two groups at 1 week or 1 year postoperatively (Fig. 2).

No patients showed mild or worse PVL as assessed by intraoperative transoesophageal echocardiography. In addition, no patients showed moderate or worse PVL at 1 week or 1 year in either group. However, three patients (3.4%) in the Avalus™ group showed mild PVL at 1 week postoperatively. Of them, two patients had an annular diameter of 19 mm and one had a type 0 bicuspid valve with an annular diameter of 23 mm. The mild PVL was sustained at 1 year postoperatively in one of these patients, whereas the remaining two patients with mild PVL were not examined at 1 year postoperatively. There was no significant difference in PVL between the two groups.

**Fig. 3:**
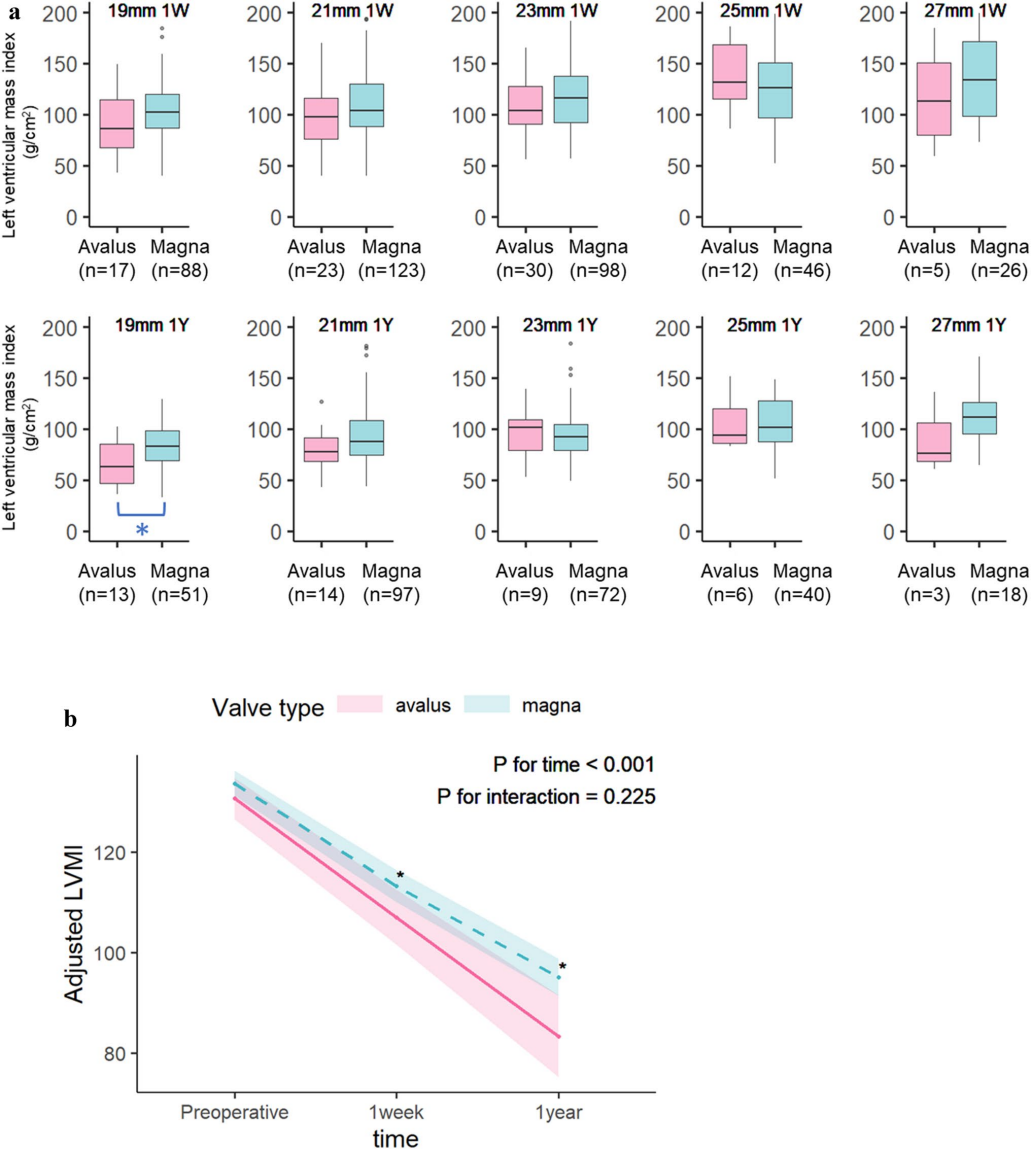
Transthoracic echocardiographic assessment of the left ventricular mass index for each valve. **a** Unadjusted (actual) data. **b** Interaction between time points (discharge, 1 year postoperatively) and valve type (Avalus™ versus Magna™)

The LVMI promptly decreased in both groups at 1 week and 1 year postoperatively and then steadily decreased in both groups. (Fig. 3). LVMI reduction appeared to predominate in the Avalus™ group compared with the Magna™ group, although there was no statistically significant difference (*p* for interactions = 0.225). After statistical adjustment, the Avalus™ group showed a significantly lower LMVI than the Magna™ group at 1 week and 1 year postoperatively.

Focusing on aortic stenosis, 46 cases of the Avalus group (53%) and 264 cases of the Magna group (69%) were statistically compared (Table 4). As a result, echocardiographic parameters were not significantly different between the two groups, apart from 1-week postoperative LVEF, which was significantly greater in the Magna group than the Avalus group.

**Table 4:** Transthoracic echocardiographic assessment in aortic stenosis patients

	Avalus	Magna	*p*
*n*	46	264	
Preoperative			
LVDd (mm)	47 [43, 53]	47 [43, 51]	0.312
LVDs (mm)	30 [27, 38]	29 [26, 34]	0.098
LVEF (%)	60.5 [51.3, 64.1]	62.2 [54.7, 67.1]	0.15
LVEF < 35%	4 (8.7)	17 (6.4)	0.53
LVMI (g/m^2^)	114 [84, 138]	120 [98, 150]	0.058
Preoperative aortic peak V (m/s)	4.5 [4.1, 4.9]	4.5 [4.0, 5.0]	0.81
Preoperative aortic mean PG (mmHg)	48 [40, 56]	49 [38, 63]	0.956
Preoperative AVA (cm^2^)	0.74 [0.63, 0.89]	0.72 [0.61, 0.85]	0.228
Preoperative AVAi (cm^2^)	0.46 [0.37, 0.54]	0.45 [0.38, 0.54]	0.77
1 week postoperatively			
Patient number	46 (100)	264 (100)	
LVDd (mm)	44.0 [38.5, 50.7]	44.0 [40.0, 49.0]	0.97
LVDs (mm)	30.0 [26.0, 38.0]	28.0 [25.0, 34.0]	0.15
LVEF (%)	52.5 [45.5, 58.0]	57.0 [49.3, 64.0]	0.01
Severe PPM	2 (4.3)	26 (10.5)	0.27
1-year postoperatively			
Patient number	27 (58.6)	178 (67.4)	
LVDd (mm)	43.0 [38.0, 46.0]	44.0 [41.0, 48.0]	0.25
LVDs (mm)	26.0 [18.0, 42.0]	27.5 [24.0, 31.0]	0.52
LVEF (%)	58.0 [23.0, 30.5]	60.0 [52.5, 65.0]	0.44
Severe PPM	0 (0.0)	15 (8.4)	0.11

Data are presented as median [interquartile range] or number (%); LVDd, left ventricular internal diameter in diastole; LVDs, left ventricular internal diameter in systole; LVEF, left ventricular ejection fraction; LVMI, left ventricular mass index; AVA, aortic valve area; AVAi, aortic valve area index; EOA, effective orifice area; EOAi, effective orifice area index; Patient-prosthesis mismatch (PPM): severe PPM means EOAi < 0.65 cm^2^/m^2^

### PPM after SAVR

Three patients (3.4%) in the Avalus™ group and in 39 patients (10.8%) in the Magna™ group (*p* = 0.12) had severe PPM at 1 week postoperatively. Of the three patients in the Avalus™ group, one patient with a 23-mm valve had a large physique (body surface area of 2.27) with an indexed EOA of 0.58. The remaining two patients had a 19-mm prosthesis implanted. None of them had PPM-related symptoms until the last follow-up. There was no significant difference in the incidence of severe PPM between the two groups for 1-year period postoperatively.

## Discussion

Compared with the PERIGON Pivotal Trial, the present study had several fundamental differences, although the early clinical outcomes were similarly good [1] 2. First, the body size was substantially smaller in our cohort than in the PERIGON study (mean body surface area of 1.6 ± 0.2 vs. 2.0 ± 0.2 m^2^, respectively). In addition, male patients predominated in the PERIGON study, while half of our patients were women. As a result, the implanted valve size was substantially smaller in our study. In fact, we implanted either a 19- or 21-mm valve in 40 patients (46%), whereas valves of these sizes were implanted in < 30% of the cohort in the PERIGON study. Generally, small stented biological valves are more difficult to implant; however, 19- or 21-mm valves were successfully implanted in our cohort using the same surgical procedure as for valves of other sizes and as for other valve prosthesis such as Magna™. This success may be attributed to the enhanced implantability of the Avalus™ valve, which is characterised by a relatively small and soft stent post and a large cuff. Although a small annulus and bicuspid valve, both of which were predominant characteristics in the study cohort, are risk factors for PVL, there were no clinically significant cases of postoperative PVL in this study. Implantation of the Avalus valve to the small aortic root needs a technical care owing to its large sewing cuff. Oversized Avalus valve should not be used in the patients having small aortic root. This suggests good implantability of the Avalus™ valve even in Japanese patients.

Importantly, the MPG and EOA of the Avalus™ valve as evaluated by echocardiography were not significantly different from those of the Magna™ valve for a 1-year period postoperatively, suggesting good functionality of the Avalus™ valve. Because the Avalus™ valve has a relatively large cuff, a concern is potential difficulty implanting a just-sized or over-sized valve, which may lead surgeons to implant an under-sized valve, particularly in patients with a small aortic annulus [8]. However, our results indicated that implanting a valve sized by the universal sizer and replica was appropriate. In addition, small valves such as the 19- or 21-mm valves produced an MPG and EOA similar to that of the Magna™ valves and resulted in good functionality in patients with a small body size.

LV hypertrophy is a risk factor for cardiac morbidity and mortality [9]. In our study, the adjusted LVMI significantly decreased over 1 year postoperatively (*p* < 0.001) (Fig. 3). Regression models including an interaction term showed that postoperative LMVI was not significantly different according to the bioprosthetic valve type (P for interaction = 0.225). Post hoc pairwise comparisons showed that the LVMI preoperatively (adjusted difference, 2.83; 95% CI  − 1.43 to 7.10; P = 0.193) and 1 week postoperatively (adjusted difference, 6.00; 95% CI  − 0.06 to 12.05; *p* = 0.052) were not significant. However, at 1 year after surgery, the LVMI was significantly lower in the Avalus™ than Magna™ group (adjusted difference, 11.52; 95% CI 2.79–20.25; *p* = 0.010). Many previous studies have identified the preoperative LVM as the most important determinant of postoperative LVM regression [10]. Other factors, such as the type and size of the implanted valve, hypertension, residual transaortic gradients, and the presence of ischemic heart disease, are thought to have an impact on LVM regression [11]. In contrast, some reports have shown that the type of prosthesis did not predict the extent of postoperative LVM regression in the long term [12], 13. The characteristic structure of the Avalus™ valve, especially the thin and flexible stent posts and leaflets, might have contributed to the difference in the LVMI after 1 year, but the cause was not clear. Follow-up surveys are required.

This study is limited by its retrospective design, small number of patients, and short follow-up period. However, we consider that this institutional report evaluating the first 87 Japanese patients enhances the understanding of this new stented bovine pericardial valve for physicians and surgeons who have just launched or are going to launch a program to implant this valve. In addition, we did not randomise the prosthetic valve selection (Avalus™ or other valves) in this study; instead, the valves were selected by individual surgeons who implanted the best-matched valve prosthesis for each patient. Because the Avalus™ valve was the primary choice of bioprosthetic valve during the study period, the study bias related to patient selection was minimal [14,15].

## Conclusion

The new stented bovine pericardial valve, Avalus™, was associated with good in-hospital outcomes and good valve functionality after SAVR in Japanese patients.
